# Sustained employability of workers in a production environment: design of a stepped wedge trial to evaluate effectiveness and cost-benefit of the POSE program

**DOI:** 10.1186/1471-2458-12-1003

**Published:** 2012-11-20

**Authors:** Berry J van Holland, Michiel R de Boer, Sandra Brouwer, Remko Soer, Michiel F Reneman

**Affiliations:** 1 Department of Rehabilitation Medicine, Center for Rehabilitation, University Medical Center Groningen, University of Groningen, Groningen, The Netherlands; 2 Department of Health Sciences, Community and Occupational Medicine, University Medical Center Groningen, University of Groningen, Groningen, The Netherlands; 3 Department of Health Sciences, Faculty of Earth and Life Sciences, Institute for Health Sciences, VU University, Amsterdam, The Netherlands; 4 Groningen Spine Center, University Medical Center Groningen, Groningen, The Netherlands

**Keywords:** Sustained employability, Functional capacity evaluation, Workers’ health surveillance, Stepped wedge trial, Production environment

## Abstract

**Background:**

Sustained employability and health are generating awareness of employers in an aging and more complex work force. To meet these needs, employers may offer their employees health surveillance programs, to increase opportunities to work on health and sustained employability. However, evidence for these health surveillance programs is lacking. The FLESH study (Functional Labour Evaluation for Sustained Health and employment) was developed to evaluate a comprehensive workers’ health promotion program on its effectiveness, cost-benefit, and process of the intervention.

**Methods:**

The study is designed as a cluster randomised stepped wedge trial with randomisation at company plant level and is carried out in a large meat processing company. Every contracted employee is offered the opportunity to participate in the POSE program (Promotion Of Sustained Employability). The main goals of the POSE program are 1) providing employee’s insight into their current employability and health status, 2) offering opportunities to improve employability and decrease health risks and 3) improving employability and health sustainably in order to keep them healthy at work. The program consists of a broad assessment followed by a counselling session and, if needed, a tailored intervention. Measurements will be performed at baseline and will be followed up at 20, 40, 60, 80, 106 and 132 weeks. The primary outcome measures are work ability, productivity and absenteeism. Secondary outcomes include health status, vitality, and psychosocial workload. A cost-benefit study will be conducted from the employers’ perspective. A process evaluation will be conducted and the satisfaction of employer and employees with the program will be assessed.

**Discussion:**

This study provides information on the effectiveness of the POSE program on sustained employment. When the program proves to be effective, employees benefit by improved work ability, and health. Employers benefit from healthier employees, reduced sick leave (costs) and higher productivity. The study can expose key elements for a successful implementation and execution of the POSE program and may serve as an example to other companies inside and outside the industry.

**Trial registration:**

The trial is registered at the Dutch Trial Register (http://www.trialregister.nl): NTR3445

## Background

Sustained health takes a prominent place in daily life and work. (Temporary) inability to work optimally due to health problems causes high productivity loss, and poses a great burden on individuals and society [1,2]. This burden is expected to increase, because of an aging workforce [3,4]. Common to a production environment are injuries and diseases related to musculoskeletal disorders (MSDs). Prevalence of musculoskeletal disorders among workers in the meat processing industry is especially high, with percentages of over 90% [5-7]. MSDs may originate from personal factors such as age, genetics, and Body Mass Index [8] or various contextual factors, such as job demands, job design, seasonality and environmental influences [9] or a combination of both.

The growing proportion of older people in the labour force stresses the need for new policies and programs to assure sustainable employability and to decrease the financial burden. Sustainable employability is defined as employees having the opportunity to perform work with preservation of health and wellbeing during their working life, now and in the future [10]. It is considered a multifactorial concept that can be assessed by multiple outcome measures. Commonly used proxies for sustained employment are work ability, productivity and absenteeism [11,12]. For instance, work ability (measured with the Work Ability Index; [13]) was observed to be a strong predictor of sustained employment which means that employees with lower reported work ability are more likely to develop health complaints and retire early [4,14].

To prevent long term sickness and work disability, risk inventory and evaluations (RI&E) have been introduced at the workplace. One of the outcomes of a RI&E is a plan to minimize potential risks for both employee and employer. In 2009 and 2010, the Dutch Labour Inspectorate performed nationwide inquiries at multiple meat processing companies. The main risk factors identified for sustained employability were related to job demands and job design (machine handling, knife handling, repetitive movements, static postures, work pressure), and contextual factors (work on platforms, biologic agents, noise, safety measures) [15,16]. Personal factors, such as age, ethnicity and employment status, were not examined, but may well form a risk factor [8].

To promote sustained employability, workers’ health surveillances (WHS) have been developed with the aim of promoting sustained employability and health, and of reducing medical costs [17]. A WHS program is developed to provide employees insight into their work ability and health status. This may offer them the opportunity to increase their probability of sustained employability and reduce potential health risks. Recently, some job-specific WHSs and job-specific interventions have been reviewed [18]. In the WHSs workers were screened on several health factors. In the interventions physical and psychological training were deployed to improve job performance. The effects of physical interventions were diverse: healthy lifestyle promotion, physical readiness training and resistance and endurance training were effective on job performance; an exercise intervention program was partly effective; respiratory muscle training was ineffective. One psychological intervention (trauma resilience training) proved to be effective on police performance. Kreis and Bodeker [19] observed positive effects of workplace health promotion, which is practically similar to a WHS, on several health risks (smoking, weight control, and physical fitness). They also observed positive cost-benefit ratios of 1:2.5 and higher for workplace health promotion programs and their effect on absenteeism. Occupational health examinations, i.e. the screening part of a WHS, including a functional capacity evaluation (FCE) have also been reviewed [20,21]. Conflicting evidence was observed for the effectiveness of occupational health examinations including FCE on the prevalence of musculoskeletal injuries [20]. These inconclusive results underscore the need for investigation and evaluation of an integral WHS program. Furthermore, an integral WHS program, including physical, mental and integrated interventions, and its effects on sustained employability has not been studied before.

The POSE program (Promotion Of Sustained Employability) was developed using elements from occupational and rehabilitation medicine. Elements from occupational medicine are e.g. WHSs, and interventions aimed at a healthy lifestyle. Elements from rehabilitation medicine are e.g. FCE, and interventions aimed at improved physical capacity. The POSE program offers employees a custom-made risk profile and, if necessary, an intervention plan using an integral approach [10]. The goal is to reduce sickness absence and, hence, reduce inflow of workers into the ‘Dutch law for Work and Income according to work ability’ (WIA), to prevent income reduction, and to increase sustained employability. The FLESH evaluation study (Functional Labour Evaluation for Sustained Health and employment) was developed to provide insight into the effectiveness of this integrated approach on several outcome measures over time.

### Objective and research questions

The purpose of the FLESH study is to evaluate the effectiveness and cost-benefit of the POSE program on work ability, productivity, and sickness absence among employees in the meat processing industry, compared to care as usual (CAU). The secondary aims are to improve perceived psychosocial and physical workload, health status, and vitality. Along with these evaluations a process evaluation will be conducted among employees and other stakeholders. The objective of this paper is to present the study protocol used in the FLESH study in which the following research questions will be addressed:

1) Does the POSE program improve the primary outcome measures work ability, absenteeism and productivity compared to CAU?

2) Does the POSE program improve the secondary outcome measures psychosocial workload, physical workload, subjective health status and vitality compared to CAU?

3) Is the effectiveness of the POSE program influenced by age, program adherence, risk category, or motivation of management?

4) To what extent do the applied methods appeal to the needs of the involved stakeholders (intervention acceptability)?

5) Is the implementation of the POSE program cost beneficial from the employers’ perspective?

## Methods/design

The CONSORT statement was used in order to describe the design of this study [22,23].

### Trial design and study participants

**Figure 1:**
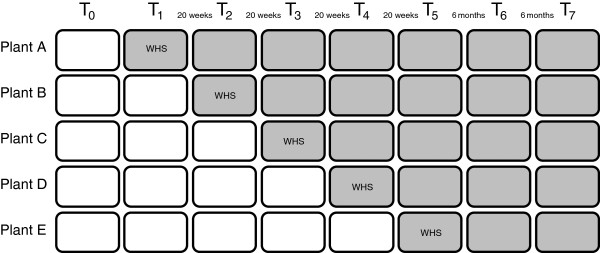
Trial design.

This study is designed as a stepped wedge trial with a follow-up of one year. The design is presented in Figure 1. The entire study will be carried out within a large meat processing company in The Netherlands, from January 2012 until August 2014.

#### Randomisation

Out of the 15 potentially available plants in The Netherlands, five plants fulfilled the inclusion criteria for the FLESH study. Inclusion criteria were having sufficient employees (n) and having budget for POSE program implementation. The order of inclusion in the study was randomly assigned.

#### Procedure

All contracted personnel (n ≈ 1000) are invited by letter to participate in the POSE program at their plant. Personnel is divided into: 1) production personnel and technical services; 2) facility services; 3) staff and office personnel.

Employees are eligible for the FLESH study when they:

– are contracted personnel.

– perform paid labour for at least 12 hours per week at the meat processing company [24].

– agree to participate in the POSE program.

– provide informed consent to participate in the study.

At T_0_, the employees at all five plants receive a baseline questionnaire. At T_1_, the POSE program is implemented at Plant A, the employees at the other plants serve as a control group and receive a follow-up questionnaire. At T_2_, the POSE program is implemented at Plant B, the program continues at Plant A, and Plant C, D and E serve as the control group, etc. T_0_ until T_5_ are separated by 20 weeks each. T_6_ and T_7_ serve as 6 months and 12 months follow-up measurements, respectively. At every time point, all employees receive a questionnaire consisting of items on the primary and secondary outcomes, as well as covariates (see Figure 1).

Because the POSE program consists of ‘care as usual’, the Medical Ethics Board of the University Medical Centre of Groningen decided that a formal ethical approval of the study protocol was not needed.

### Intervention

**Figure 2:**
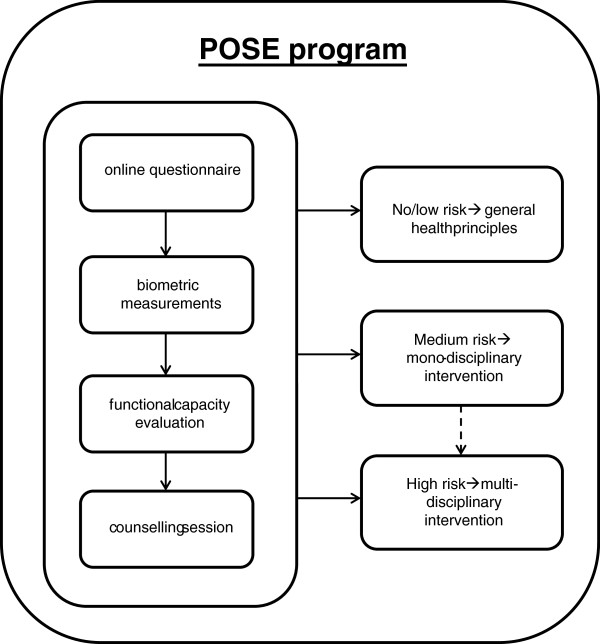
Design of the POSE program within the company.

Before the start of the POSE program, employees provide their informed consent for participating in the study. The POSE program consists of assessments and a counselling session. When individual results indicate an increased risk for health problems or absence, an intervention program is offered. An overview of the POSE program is provided in Figure 2.

#### Digital questionnaire

Employees are asked to complete an electronic questionnaire before the start of the POSE program. This questionnaire focuses on work ability, health and lifestyle. Employees with limited understanding of the Dutch language are assisted in filling out the questionnaire.

#### Biometric measurements

Standard biometric measurements are performed on the following parameters: body length, weight, fat percentage, waist circumference, blood pressure, lung function, and cholesterol and glucose levels. Also a hearing test and visual test are administered. These measurements are performed by a physician assistant (PA).

#### Functional capacity evaluation

Before the start of the FCE, employees fill out the Physical Activity Readiness Questionnaire (PAR-Q) [25]. If at least one question is answered positively, employees are not allowed to perform the tests of the FCE that involve peak aerobic and strength capacity. Employees are also not allowed to perform these tests when resting systolic blood pressure exceeds 159 mmHg or resting diastolic blood pressure exceeds 100 mmHg [26]. Depending on the job of an employee, a FCE is performed adjusted to the employee’s job demands. Five domains of functional capacity (material handling, postural tolerance, coordination and repetition, hand and finger strength, and energetic capacity) are tested. Exact procedures have been described elsewhere [26]. The sub maximal Åstrand cycle ergometer test is used to evaluate energetic capacity [27]. The FCE is administered by registered vocational physiotherapists. The physiotherapists receive a one-day training by a licensed trainer, specifically for this study.

#### Counselling session

The employee receives feedback on his/her results from the assessments by a consultant (in most cases the physiotherapist) and advice on whether or not to take consecutive actions. When an employee is advised to take consecutive actions he/she is encouraged to enrol in one of the pre-specified interventions. Depending on the outcome of the assessments, employees are categorised into three different risk profiles threatening sustainable employability: 1) no or very low risk, 2) low to medium risk, and 3) high risk. These risk profiles are based on guidelines by the Netherlands Society of Occupational Medicine (NVAB) [28-32] and guidelines on cardiovascular risk management [33]. Depending on the risk profile, no intervention, a monodisciplinary or multidisciplinary intervention is advised. For instance, multidisciplinary interventions are advised when sick-leave is involved and musculoskeletal and mental complaints are present.

1) For persons with no or very low risk, meetings focussing on primary prevention are organised where general health principles are presented. The meetings are organised at the company’s plants. During these meetings, several topics are addressed, such as lifestyle (change), workload, work capacity and adjustment latitude. Employees are pointed to organisational activities such as company fitness and canteen policy.

2) Persons with low to medium risk are either offered a monodisciplinary intervention or referred to their general practitioner (GP). The intervention can be deployed by a physiotherapist, a psychologist or a dietician. The choice of discipline and the contents of the intervention depend on the outcomes of the assessments, e.g. physiotherapy in case of MSDs. Basically, the interventions are aimed at preserving or restoring functional capacity of the employee, but they can also be aimed at the (work) environment, i.e. at creating and utilizing adjustment latitude. In the present study three ‘physical’ interventions and two ‘non-physical’ interventions are offered: improving personal work capacity, reducing and preventing back complaints, reducing and preventing neck and shoulder complaints, guidance by a dietician in case of substantial overweight of an employee, and stress reduction. These interventions were based on evidence described in several reviews [34-37].

3) Employees with more complex or multiple complaints, who are absent from work regularly or continuously, are offered a multidisciplinary intervention. These multidisciplinary rehabilitation (MR) programs are conducted by professionals from several disciplines, such as a rehabilitation physician, physiotherapists, occupational therapists, and psychologists. Disciplines are deployed on indication. MR is deemed the most preferred evidence-based intervention for chronic complaints of the musculoskeletal system [38]. MR also integrates interventions to adjust the workload, such as job control possibilities.

### Care as usual

Regular healthcare, unrelated to the WHS program, is considered care as usual. Programs already running within the company are also considered CAU.

### Outcomes

#### Effect evaluation

The primary outcomes in this study are work ability, productivity and absenteeism.

– Work ability is measured with the Work Ability Index (WAI) [13]. The WAI consists of 28 questions focusing on mental and physical work ability, injuries and diseases, and future expectations. A sum score is calculated, ranging from 7–49.

– Productivity at the individual level is measured by self-report using the Quality and Quantity (QQ) questionnaire [39]. The QQ addresses the quality and quantity of work during the previous workday with two questions, each to be answered with an 11-point Numeric Rating Scale (NRS).

– Absenteeism data are provided by the occupational health service (OHS).

The secondary outcomes are psychosocial and physical workload, health status, and vitality.

– Several aspects of the psychosocial workload are measured by the second version of the Copenhagen Psychosocial Questionnaire (COPSOQ II) [40]. These aspects are quantitative work demands, work pace, autonomy, possibilities for development, meaning of work, job satisfaction, social support from supervisor, social support from colleagues, social relationships among colleagues, and sense of community. This set of concepts comprises 19 items. Reproducibility of the Dutch language version of the COPSOQ has been shown to be adequate to good [41].

– Self-reported health status is evaluated by the Dutch version of the EuroQol-5D [42]. It consists of five short questions on various health domains (e.g. mobility) and a health thermometer (0–100). The valuation of EQ-5D scores is based on the Dutch tariff, and results in scores between 0 (dead) and 1 (completely healthy).

– Self-reported vitality is assessed by a subscale of the RAND-36 questionnaire [43]. This scale consists of four questions on a 5-point Likert scale. The RAND-36 is a highly reliable instrument with satisfactory construct validity [44].

#### Covariates

Personal and work characteristics are assessed as covariate in this study.

– Most personal and work characteristics (e.g. age, gender, commuting time) are obtained through the questionnaire. Additional personal and work characteristics (e.g. contract hours, function years) are retrieved from the company headquarters.

– To assess physical workload, movement, and posture during work, a short version of the Dutch Musculoskeletal Questionnaire (DMQ) is administered [45]. The 21 items of the DMQ focus on repetitive movement, awkward postures, and force components. Validity has been shown to be sufficient [45].

#### Cost-benefit evaluation

The cost-benefit of the POSE program will be evaluated from the employers’ perspective. The cost-benefit analysis will evaluate the total costs of the POSE program for the company and will compare absenteeism costs before and after the POSE program. Costs (in Euros) include:

– Costs of the POSE program, which are the costs of the program itself and the sequential costs of company interventions (excluding insured interventions).

– Costs due to time spent by employees on the different elements of the POSE program (questionnaire, assessments, counselling, interventions), which results in lost productivity.

– Absenteeism costs. These data are obtained from the OHS’s registry system. The number of absence days will be expressed in monetary terms.

– Productivity loss at work which will be assessed using the quantity scale of the QQ questionnaire [39], ranging from 0 (nothing) to 10 (regular quantity).

– Replacement costs associated with employee turnover will be calculated as the sum of the costs for hiring and training a new employee.

#### Process evaluation

A process evaluation will be conducted to evaluate the preparation, implementation, execution, and follow-up of the POSE program according to a number of key components [46,47]. Context (organisational and environmental factors that affect the intervention), recruitment (procedures to recruit employees for the POSE program), and reach (attendance rates of employees) will be evaluated at the level of company management. Information on dose delivered (the amount of program components actually delivered by the providers), dose received (the extent to which employees make use of recommended program components), and fidelity (the extent to which the program was delivered as planned) will be obtained from the OHS. This evaluation will provide deeper insight into components that make the POSE program a failure or a success [48,49]. Furthermore, the satisfaction with the POSE program will be evaluated among participating employees. For that purpose an adapted version of the Client Satisfaction Questionnaire (CSQ-8) [50] and Europep Questionnaire [51] will be sent to all participants nine months after the initiation of the program.

### Sample size

For the sample size calculation, we used the formula proposed by Hussey and Hughes for stepped wedge cluster randomised trials [52]. A pilot study among 116 employees from two plants resulted in a standard deviation (SD) of 7.0 on the WAI and an Intraclass Correlation Coefficient (ICC) for within location clustering of 0.034. Using these figures and assuming a two-sided alpha of 0.05, we calculated that we needed 4 locations with 44 employees each in order to have a 0.90 power to detect a difference of 3 points on the WAI between intervention and CAU. A sensitivity analysis in which we assumed an ICC of 0.10 resulted in four plants with 46 employees each, which is in accordance with the notion that the sample size needed for a stepped wedged trial is relatively insensitive to the ICC [52]. As described under trial design, we will include five plants in the study in order to overcome any potential problems in the inclusion and follow-up of the employees.

### Blinding

Participants, care providers, and researchers are not blinded for the group assignment.

### Statistical analyses

#### POSE program

The effect of the trial on the primary and secondary outcomes will be analysed using linear (for the continuous outcomes) and logistic (for the binary outcomes) multi-level analyses including a random coefficient for plant and a fixed effect for time. The latter parameter is included to adjust for any potential time effects [52]. All tests will be performed two-sided, assuming an alpha of 0.05.

#### Cost-benefit

The effect of the trial on the costs will also be analyzed using linear multi-level analyses including a random coefficient for plant and a fixed effect for time. Bootstrapping will be used in case the costs will turn out to follow a positively skewed distribution.

## Discussion

This paper presents the design of a stepped wedge trial to evaluate the effectiveness of a comprehensive workers’ health surveillance program in a meat production environment. The POSE program aims to balance the functional capacities of employees with work demands. Detecting and restoring imbalances between capacities and demands in an early stage may contribute to sustained employability.

The current state of WHS programs and the use of FCEs in employment examinations have recently been reviewed [20,21]. The authors observed no evidence for an association between the length of FCEs and injury relapse. They also observed that general health examinations did not lead to decreased sickness absence, but did lead to more rejected job applications. Furthermore, they observed that job-specific pre-employment examinations lead to reduced occupational disease, injury or sickness absence. The effectiveness of pre-employment examinations, including FCE, on musculoskeletal injuries could not be demonstrated. On the other hand, several individual studies have demonstrated the predictive value of FCE on sustainable return to work [53], risk for future work disability [54] and claim closure / benefit suspension [55,56]. Better FCE performance was associated with better outcomes, i.e. earlier return to work, lower risk for future work disability and earlier claim closure. In the above mentioned studies WHS and FCE results were used in association with work outcomes, not as a screener to deploy tailor-made interventions. No studies were identified reporting on WHS, FCE and integrated interventions, which underpins the need for this study.

### Strengths

To our knowledge this is the first study that evaluates a WHS consisting of multiple assessments, i.e. a questionnaire, biometric assessments, and FCE, and that also deploys tailor-made interventions.

The design of our study has some benefits over a parallel arm randomised controlled trial. The design allows to perform a within group and between group analysis, i.e. each plant serves as its own control, but also serves as control for the other plants. This limits the risk of confounding and increases statistical power.

A strength of this study is the expected high participation rate of the POSE program. During a pilot study at two locations (not included in this study), the participation rate was 80-90%. We expect the same participation rates within our study. We strive to reach this participation throughout the entire study period. During the follow-up measurements we expect this rate to decrease over time. A careful distribution process and clear communication about the follow-up questionnaires might mitigate this decrease.

When the POSE program proves to be effective within the company, we expect that the results of this trial can be used by other companies in the sector, which employ approximately 20,000 people in The Netherlands. Especially, since multiple plants are included in this study, this provides more generalisable results for all production workers. This WHS program was job-specific, so perhaps results cannot be generalised directly to other sectors. However, it is reasonable to presume that a custom-made WHS program can be adapted to other related sectors with similar results.

### Limitations / weaknesses / risk of bias

We have not included translations of our questionnaire for foreign employees, whose Dutch language skills may be insufficient to complete the questionnaire. In the Dutch meat processing industry, about 25% of the employees is born outside The Netherlands [15]. We strive to assist those employees in filling out the questionnaires, but this will probably not be feasible in some cases. This issue may therefore limit the generalisability of our results. Another issue that might have impact on the generalisability of the findings is the fact that temporary production workers could not be included in the study, because of practical reasons. These workers are common in the meat processing industry (approx. 30%) and many are foreign [15,16]. Thus, a substantial part of all workers in this study will expectedly not participate, which limits generalisation towards these groups.

The company has already been working on absenteeism reduction for a few years before implementing the POSE program. In these years, sickness absence has dropped from 7.0% to 4.5%. It is unknown whether this may lead to floor effects. Possible effects of the POSE program might therefore go unnoticed. Perhaps it is not realistic to expect absenteeism rates to drop even further, due to the type of work.

Plants where the POSE program has not yet been implemented could already perform health related activities prior to implementation of the POSE program. Such activities might influence the results of the POSE program and therefore influence possible effects of the POSE program. We emphasized that the company should not start health-related activities prior to POSE program implementation, other than care as usual activities.

### Scientific relevance

Within our study an integrated approach is deployed. The study will yield valuable information on the effectiveness of this approach on work related outcomes. Combining the results of the process evaluation and the intervention effects will hopefully provide insight into the effectiveness of separate aspects, and perhaps underlying reasons for the effectiveness.

### Practical application

The results of this study will provide information on the effectiveness of the POSE program on sustained employment. When the program proves to be effective, employees will benefit from this by an improved health and perhaps a healthier working environment. Employers might benefit from healthier employees, and in the case the intervention proves to be cost-beneficial, reduced costs and higher productivity. Furthermore it will expose key elements for a successful implementation and execution of the POSE program and may serve as an example to other companies inside and outside the industry.

## Abbreviations

CAU: Care as usual; CONSORT: CONsolidated standards of reporting trials; COPSOQ II: Copenhagen psychosocial questionnaire 2^nd^ version; CSQ-8: Client satisfaction questionnaire; DMQ: Dutch musculoskeletal questionnaire; FCE: Functional capacity evaluation; FLESH: Functional labour evaluation for sustained health and employment; GP: General practitioner; ICC: Intraclass correlation coefficient; MR: Multidisciplinary rehabilitation; MSD(s): Musculo skeletal disorder(s); NVAB: Netherlands society of occupational medicine (nederlandse vereniging voor arbeids- en bedrijfsgeneeskunde); OHS: Occupational health service; PAR-Q: Physical activity readiness questionnaire; POSE: Promotion of sustained employability; QQ: Quality and quantity questionnaire; RI&E: Risk Inventory and Evaluation; WAI: Work ability index; WHS: Workers’ health surveillance; WIA: Dutch law for work and income according to work ability.

## Competing interests

All authors declared that they have no competing interest.

## Authors’ contributions

RS, MR and SB developed the initial study protocol and acquired funding for the FLESH study. They also provided valuable input to this design paper, which was drafted by BvH, MdB, and RS. All authors commented on the draft versions. All authors read and approved the final manuscript.

## Pre-publication history

The pre-publication history for this paper can be accessed here:

http://www.biomedcentral.com/1471-2458/12/1003/prepub
